# LRRK2 Regulates CPT1A to Promote β-Oxidation in HepG2 Cells

**DOI:** 10.3390/molecules25184122

**Published:** 2020-09-09

**Authors:** Chiao-Wei Lin, Yu-Ju Peng, Yuan-Yu Lin, Harry John Mersmann, Shih-Torng Ding

**Affiliations:** 1 Institute of Biotechnology, National Taiwan University, Taipei 106, Taiwan; d04642002@ntu.edu.tw; 2 Department of Animal Science and Technology, National Taiwan University, Taipei 106, Taiwan; d98626001@ntu.edu.tw (Y.-J.P.); yylin@ntu.edu.tw (Y.-Y.L.); mersmann@msn.com (H.J.M.)

**Keywords:** LRRK2, β-oxidation, CPT1A, NAFLD

## Abstract

Leucine-rich repeat kinase 2 (LRRK2) is involved in lipid metabolism; however, the role of LRRK2 in lipid metabolism to affect non-alcoholic fatty liver disease (NAFLD) is still unclear. In the mouse model of NAFLD induced by a high-fat diet, we observed that LRRK2 was decreased in livers. In HepG2 cells, exposure to palmitic acid (PA) down-regulated LRRK2. Overexpression and knockdown of LRRK2 in HepG2 cells were performed to further investigate the roles of LRRK2 in lipid metabolism. Our results showed that β-oxidation in HepG2 cells was promoted by LRRK2 overexpression, whereas LRRK2 knockdown inhibited β-oxidation. The critical enzyme of β-oxidation, carnitine palmitoyltransferase 1A (CPT1A), was positively regulated by LRRK2. Our data suggested that the regulation of CPT1A by LRRK2 may be via the activation of AMP-activated protein kinase (AMPK) and peroxisome proliferator-activated receptor α (PPARα). The overexpression of LRRK2 reduced the concentration of a pro-inflammatory cytokine, tumor necrosis factor α (TNFα), induced by PA. The increase in β-oxidation may promote lipid catabolism to suppress inflammation induced by PA. These results indicated that LRRK2 participated in the regulation of β-oxidation and suggested that the decreased LRRK2 may promote inflammation by suppressing β-oxidation in the liver.

## 1. Introduction

Non-alcoholic fatty liver disease (NAFLD) is a common chronic liver disease with a global prevalence [1]. NAFLD is caused by excess fat accumulation in livers [2]. About 20–25% of NAFLD patients progress to non-alcoholic steatohepatitis (NASH), a severe form of NAFLD with inflammation [3]. NASH patients have a high risk of cirrhosis and hepatocellular carcinoma [1,3,4]. Obesity, one of the primary risk factors for NAFLD [1,2,5], leads to the efflux of free fatty acids from adipose tissues into circulation [6,7] resulting in the influx of free fatty acids into other organs, such as liver [8]. Palmitic acid (PA, 16:0), a fatty acid abundant in plasma [9], is increased in the plasma of NAFLD patients [10,11]. PA can cause apoptosis [12,13], inflammation [14,15], and the impairment of fatty acid oxidation [16,17]. These factors may contribute to the development of NAFLD [18,19]. However, the mechanism of the harmful effects caused by PA has not been fully understood.

The accumulated free fatty acid in cells can be converted to lipotoxic lipids which have been suggested as a contributor to the inflammation of hepatocytes [20]. Carnitine palmitoyltransferase 1A (CPT1A), an enzyme in the outer membrane of mitochondria, functions to transport fatty acid from the cytosol into mitochondria [21]. After transport into mitochondria, the intracellular non-esterified fatty acids (NEFA) can be catabolized via β-oxidation within mitochondria [22,23]. CPT1A can be up-regulated via the activation of peroxisome proliferator-activated receptor α (PPARα) [24,25] and AMP-activated protein kinase (AMPK) to promote β-oxidation [26,27,28,29]. CPT1A can promote β-oxidation to decrease the intracellular free fatty acid and suppress the secretion of pro-inflammatory cytokines, such as tumor necrosis factor α (TNFα) [30,31]. Therefore, enhancing β-oxidation may be a strategy to suppress inflammation in NAFLD.

Leucine-rich repeat kinase 2 (LRRK2) is a multifunctional protein with kinase, GTPase, and protein interaction domains [32,33]. Due to its multiple domains, LRRK2 can associate with various proteins to participate in different functions, including mitochondrial homeostasis, inflammation, autophagy, and vesicle-trafficking [33,34]. In humans, LRRK2 is associated with Parkinson’s disease, inflammatory bowel disease, and leprosy [35,36,37]. In addition, possible LRRK2 involvement in the regulation of lipid metabolism has been shown in LRRK2 knockout rodents that displayed an accumulation of lipids in livers and kidneys [38,39]. The level of ceramide, a lipotoxicity-causing lipid derivative, is significantly higher in the LRRK2 knockout compared to wild-type mice [40]. The elevation of ceramide may be induced by glucocerebrosidase 1 activity, an enzyme that converts glucosylceramide to ceramide and glucose [40]. The Y1699C mutant of LRRK2, a pathogenic mutant for Parkinson’s disease, promotes the enlargement of lipid droplets through the phosphorylation of Rab8a in an adipose cell line [41].

Previous studies suggest that LRRK2 may play roles in the regulation of lipid metabolism. However, the detailed mechanism is still unclear. In the current paper, we observed the change of LRRK2 expression in NAFLD livers accompanied by inflammation in mice. Furthermore, we investigated the roles of LRRK2 in lipid metabolism in vitro. Our results suggested that LRRK2 plays a role in the regulation of β-oxidation. Moreover, the down-regulation of LRRK2 in the livers of NAFLD mice may result in the suppression of β-oxidation, which may result in inflammation.

## 2. Results

### 2.1. LRRK2 Was Down-Regulated in the Liver of High-Fat Diet Induced NAFLD Mice

**Figure 1:**
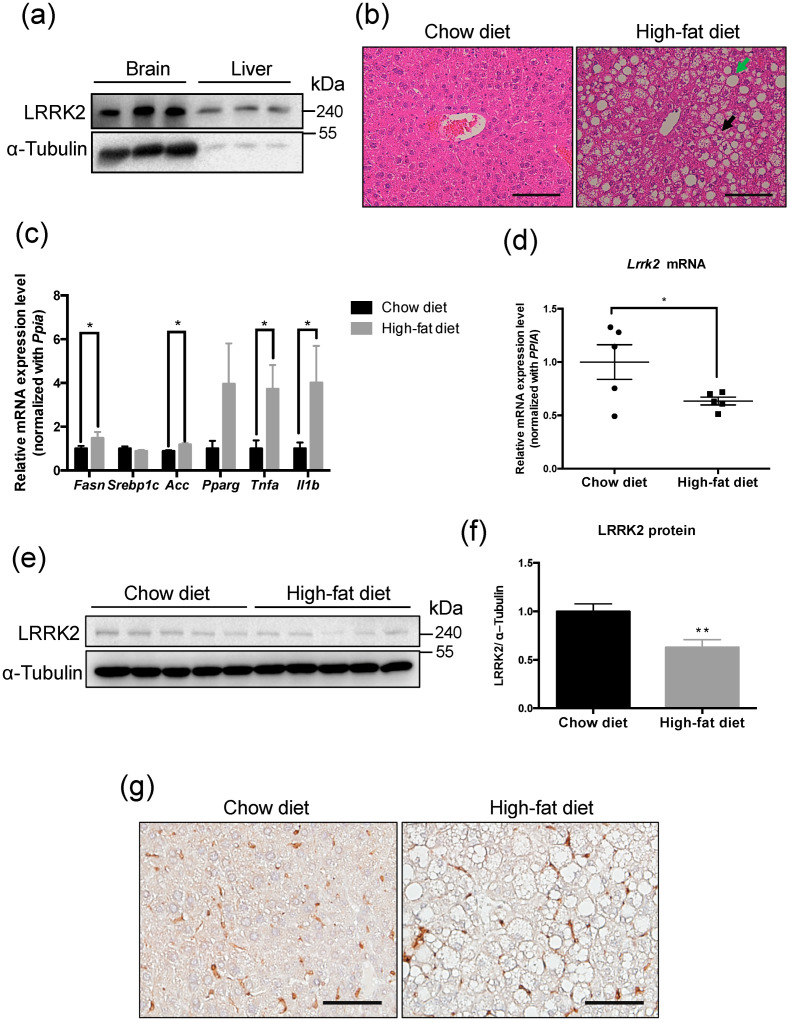
Leucine-rich repeat kinase 2 (LRRK2) was down-regulated in the liver of high-fat diet induced non-alcoholic fatty liver disease (NAFLD) mice. (**a**) A western blot was used to detect the presence of LRRK2 in an equal amount (25 μg) of lysates from brains and livers. (**b**) Histological analysis of the livers in the mice fed with indicated diets by H&E staining. Green arrow indicates steatosis, and black arrow indicates hepatic ballooning. Scale bar = 50 μm. (**c**) The lipogenesis related genes, *Fasn*, *Srebp1c*, *Acc*, and *Pparg*, and pro-inflammatory cytokine genes, *Tnfa* and *Il1b*, were analyzed in the livers of the mice fed with a chow diet or a high-fat diet using real-time PCR. (**d**) Relative levels of LRRK2 mRNA in the livers of chow diet and high-fat diet groups were analyzed by real-time PCR. (**e**) The protein expression of LRRK2 in the livers of chow diet and high-fat diet groups was analyzed by western blot. (**f**) The quantitative results of protein levels of LRRK2 in western blots. (**g**) IHC analysis of the livers in indicated groups using anti-LRRK2 antibody. Scale bar = 50 μm. The quantitative data are shown as mean ± SEM (*n* = 5 for each group). T-test, * *p* ≤ 0.05, ** *p* ≤ 0.01.

To confirm the presence of LRRK2 expression, brain and liver lysates from C57BL/6JNarl male mice were analyzed. The result showed that LRRK2 protein was present in the liver and brain tissues (Figure 1a). To investigate the association of LRRK2 and lipid metabolism in the liver, the NAFLD mouse model was induced by high-fat diet feeding for 16 weeks. After histological examination by hematoxylin and eosin (H&E) staining, livers from mice fed a high-fat diet displayed hepatic steatosis and ballooning (Figure 1b). The lipogenesis-related genes, *Fasn* and *Acc*, and pro-inflammatory genes, *Tnfa* and *Il1b*, were up-regulated (*p* < 0.05) in the livers of NAFLD mice (Figure 1c). The high-fat diet induced NAFLD mice presented characteristics of NASH [42,43]. In addition, current data showed both that the change in gene and protein expression of LRRK2 were observed in livers of NAFLD mice. The mRNA expression of LRRK2 was decreased (*p* < 0.05) in NAFLD livers compared to livers of the control group by real-time PCR (Figure 1d). The LRRK2 protein was lower (*p* < 0.05) in the NAFLD livers compared to the control group (Figure 1e,f). The results of immunohistochemistry (IHC) analysis confirmed that LRRK2 was down-regulated in the liver of NAFLD mice (Figure 1g).

### 2.2. Palmitic Acid Reduced the Expression of LRRK2 in HepG2 Cells

**Figure 2:**
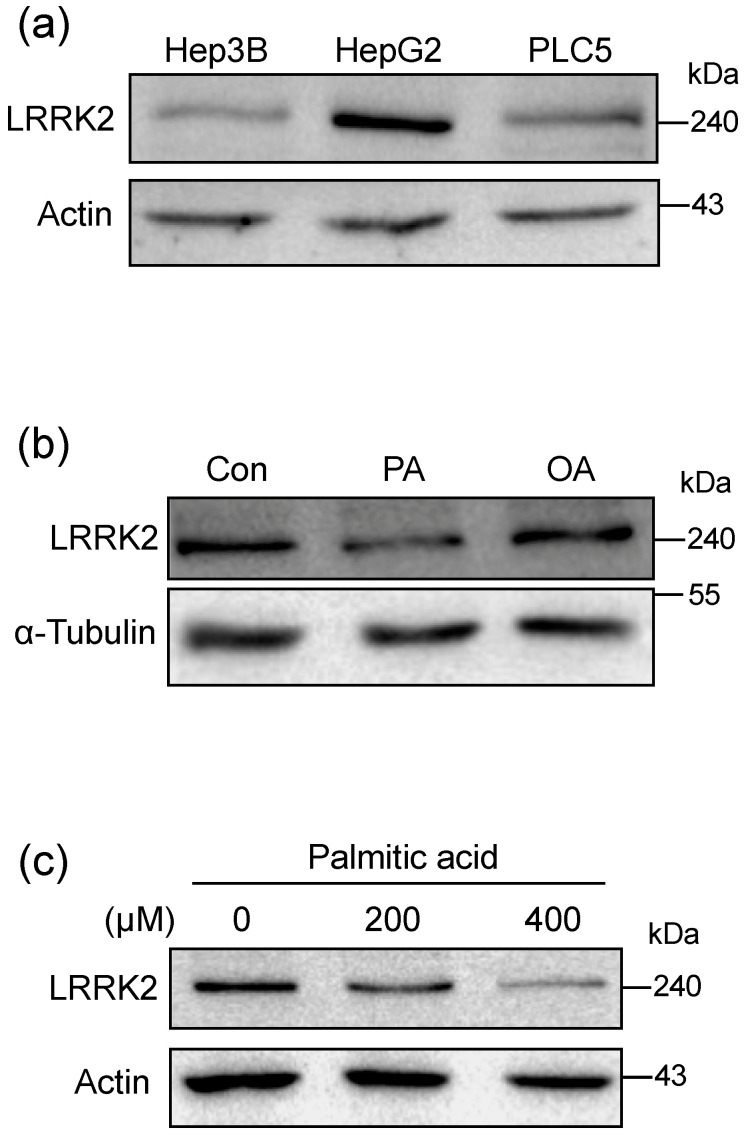
Palmitic acid reduced the expression of LRRK2 in HepG2 cells. (**a**) Western blots were used to detect the presence of LRRK2 in the liver cell lines, Hep3B, HepG2, and PLC5. (**b**) A western blot was used to analyze the levels of LRRK2 protein in HepG2 cells treated with vehicle, 400 μM PA, or 400 μM OA, respectively. HepG2 cells were treated with indicated fatty acids for 24 h. (**c**) Protein levels of LRRK2 in the HepG2 cells treated with PA at indicated concentrations for 24 h and analyzed by western blot.

To determine whether LRRK2 is expressed in liver cell lines, we performed a western blot to examine the protein expression of LRRK2 in various liver cell lines, including Hep3B, HepG2, and PLC5. The result showed that LRRK2 was detectable in all the aforementioned liver cell lines (Figure 2a). The level of LRRK2 in HepG2 cells was higher than in Hep3B and PLC5 cells (Figure 2a). Therefore, we used HepG2 cells as a cellular model for further experiments. After treatment with 400 μM oleic acid (OA, C18:1) for 24 h, the level of LRRK2 was comparable to that in the control group (Figure 2b). However, after treatment with 400 μM palmitic acid (PA, C16:0) for 24 h, the level of LRRK2 was decreased in HepG2 cells (Figure 2b). The decrease in LRRK2 with PA treatment was in a dose-dependent manner (0, 200, and 400 μM) (Figure 2c). These results indicated that the level of LRRK2 was down-regulated by PA treatment of HepG2 cells.

### 2.3. Overexpression of LRRK2 Promoted Catabolism of Free Fatty Acid in PA-Treated HepG2 Cells

**Figure 3:**
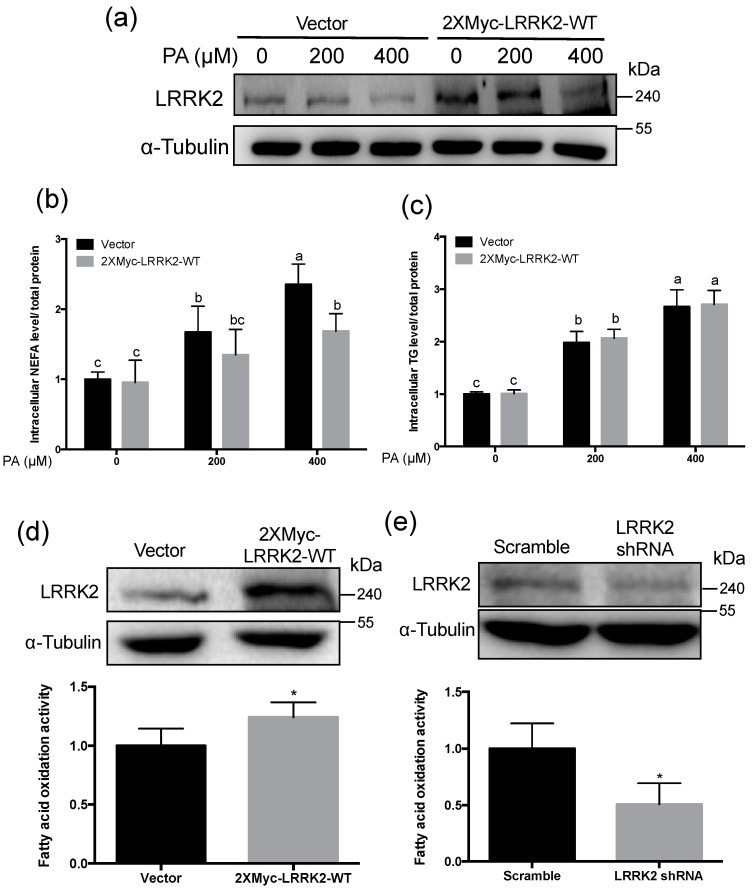
Overexpression of LRRK2 promoted catabolism of free fatty acid in palmitic acid (PA)-treated HepG2 cells. (**a**) The HepG2 transfect with 2XMyc-LRRK2-WT plasmid was as LRRK2-overexpressed group. The HepG2 cells transfected with control plasmid was as vector control. Both the control and the LRRK2- overexpressed HepG2 cells were treated with PA at 0, 200 or 400 μM, respectively. (**b**) The levels of intracellular NEFA (*n* = 5 for each group) and (**c**) intracellular TG (*n* = 3 for each group) were measured. The levels of intracellular NEFA and TG were normalized with the protein concentration of the individual lysate. Data are shown as mean ± SD. Two-way ANOVA, * *p* ≤ 0.05. Groups with no significant difference were labeled with a common letter. (**d**) The cell lysates from control and LRRK2-overexpressed HepG2 cells were used to analyze the activity of fatty acid oxidation. (*n* = 5 for each group) Data are shown by mean ± SD. T-test, * *p* ≤ 0.05. A western blot was used to present the levels of LRRK2 in vector control and LRRK2-overexpressed HepG2 cells. (**e**) The HepG2 cells with shRNA targeting to LRRK2 was as LRRK2- knockdown group. The HepG2 cells with shRNA having no hits in any human mRNAs was as a scramble control group. The cell lysates from scramble control and LRRK2-knockdown (KD) HepG2 cells were used to analyze the activity of fatty acid oxidation. (*n* = 4 for each group) Data are shown as mean ± SD. T-test, * *p* ≤ 0.05. A western blot was used to present the levels of LRRK2 in scramble-control and LRRK2-knockdown HepG2. Vector: vector control; Scramble: scramble control.

To examine whether LRRK2 plays a role in lipid metabolism, we generated LRRK2-ovexpressed HepG2 cells via transfection with the 2XMyc-LRRK2-WT plasmid [44], harboring a wild-type LRRK2 gene, into HepG2 cells. The control group was the HepG2 cells transfected with the control vector. To investigate the role of LRRK2 in lipid metabolism, control and LRRK2-overexpressed HepG2 cells were treated with PA at 0, 200, and 400 μM for 24 h (Figure 3a). The NEFA in control HepG2 cells was increased (*p* < 0.05) after PA treatment (Figure 3b). After treatment with 400 μM PA, the level of NEFA in LRRK2-overexpressed HepG2 was lower (*p* < 0.05) than in the control group (Figure 3b). However, the level of triglyceride (TG) in LRRK2-overexpressed HepG2 cells was comparable to the control group after treatment with PA, at 0, 200, and 400 μM for 24 h. (Figure 3c). These data suggested that LRRK2 may play a role in the catabolism of NEFA, but not TG. The intracellular NEFA can be transported into mitochondria and degraded through β-oxidation [23]. The overexpression of LRRK2 decreased the levels of intracellular NEFA after PA treatment; this may result from the enhancement of β-oxidation in HepG2 cells. To examine whether LRRK2 is involved in the regulation of β-oxidation, we evaluated the activity of fatty acid oxidation in the control and LRRK2-overexpressed HepG2 cells. The data showed that the activity of fatty acid oxidation was increased (*p* < 0.05) in LRRK2-overexpressed HepG2 cells compared to the control group (Figure 3d). In contrast, the activity of fatty acid oxidation was decreased (*p* < 0.05), while LRRK2 was knocked-down by short hairpin RNA (shRNA) (Figure 3e). These data indicated that LRRK2 played a role in the free fatty acid catabolism.

### 2.4. LRRK2 Positively Regulated CPT1A in HepG2 Cells

**Figure 4:**
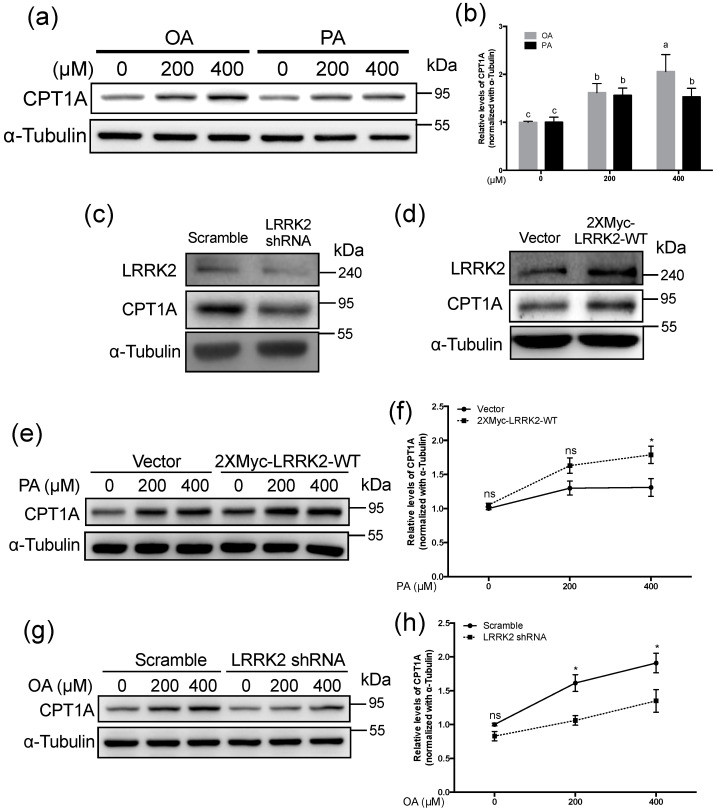
LRRK2 positively regulated carnitine palmitoyltransferase 1A (CPT1A) in HepG2 cells. (**a**) Western blots were used to analyze the levels of LRRK2 in HepG2 cells after treatment with 0, 200 or 400 μM OA or PA for 24 h. (**b**) The quantified data for the LRRK2 protein levels in HepG2 cells treated with the indicated concentrations of OA or PA. (*n* = 5 for each group) Data are shown as mean ± SD. Two-way ANOVA, * *p* ≤ 0.05. Groups with no significant difference were labeled with a common letter. (**c**) A western blot was used to analyze the protein levels of LRRK2, CPT1A and α-Tubulin in the HepG2 cells with scramble shRNA or LRRK2 shRNA and (**d**) the HepG2 cells transfected with vector control or 2XMyc-LRRK2-WT plasmid. (**e**) The levels of CPT1A after treatment with 0, 200 or 400 μM of PA for 24 h in the vector control and LRRK2-overexpressed HepG2 cells. (**f**) The levels of CPT1A were quantitated and normalized with its levels of α-Tubulin (*n* = 4 for each group). All data were further normalized to the group with 0 μM of PA. Data are shown as mean ± SD. Two-way ANOVA, * *p* ≤ 0.05, ns: non-significant difference. (**g**) The levels of CPT1A after treatment with 0, 200 or 400 μM of OA for 24 h in the scramble control and LRRK2-knockdown HepG2 (detected using a western blot). (**h**) The levels of CPT1A were quantified and normalized to the sample levels of α-Tubulin (*n* = 4 for each group). All the data were further normalized to the group with 0 μM of OA. Data were shown as mean ± SD. Two-way ANOVA, * *p* ≤ 0.05, ns: non-significant difference. Vector: vector control; Scramble: scramble control.

β-oxidation is a critical process of fatty acid catabolism and it can be disrupted by PA [16]. Our data showed that PA led to the down-regulation of LRRK2 in HepG2 cells, whereas treatment with OA showed no effects on the protein expression of LRRK2. To compare the effects of OA and PA treatments on β-oxidation, we examined the levels of CPT1A, a rate limiting enzyme of β-oxidation, after treatment with OA or PA at 0, 200, and 400 μM for 24 h in HepG2 cells. The data showed that CPT1A was increased (*p* < 0.05) by both OA and PA treatment (Figure 4a,b). However, the level of CPT1A stimulated by 400 μM PA was lower (*p* < 0.05) than that stimulated by OA (Figure 4a,b). To confirm whether LRRK2 is involved in the regulation of CPT1A, we analyzed the expression levels of CPT1A in HepG2 cells with the manipulation of the expression of LRRK2. Our data showed knockdown of LRRK2 decreased CPT1A in HepG2 cells (Figure 4c). Conversely, overexpression of LRRK2 increased the levels of CPT1A in HepG2 cells (Figure 4d). Previous studies indicate that fatty acid induces β-oxidation by increasing CPT1A [23,45,46]. Therefore, we analyzed the changes of CPT1A in the LRRK2-overexpressed and control HepG2 cells after treatment with PA at 0, 200, or 400 μM. Consistent with previous studies [45,46], both the control and LRRK2-ovexexpressed HepG2 cells had increased CPT1A after treatment with 200 or 400 μM PA for 24 h (Figure 4e). After treatment with 400 μM PA, the level of CPT1A was higher (*p* < 0.05) in LRRK2-overexpressed HepG2 cells than in control cells (Figure 4f). We analyzed the change in CPT1A in control and LRRK2-knockdown HepG2 cells after treatment with OA (caused no decrease of LRRK2). After treatment with 200 or 400 μM OA for 24 h, the level of CPT1A was increased in control and LRRK2-knockdown HepG2 cells (Figure 4g). After treatment with of 400 μM OA, the level of CPT1A was lower (*p* < 0.05) in LRRK2-knockdown cells than in control cells (Figure 4h). These data indicated that LRRK2 plays a role in the regulation of CPT1A.

### 2.5. LRRK2 Activated AMPK and PPARα in HepG2 Cells

**Figure 5:**
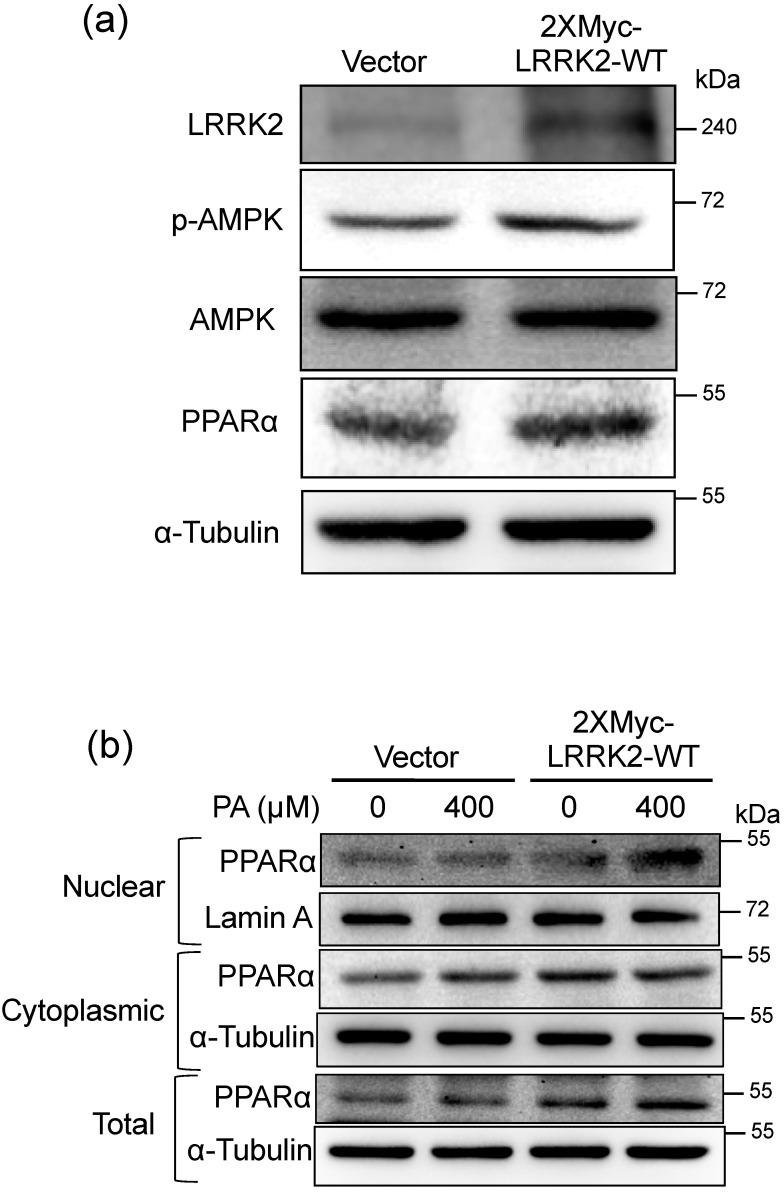
LRRK2 activated AMP-activated protein kinase (AMPK) and peroxisome proliferator-activated receptor α (PPARα) in HepG2 cells. (**a**) Western blots were used to analyze the protein levels of LRRK2, p-AMPK, AMPK, PPARα, and α-Tubulin in the HepG2 cells transfected with vector control or 2XMyc-LRRK2-WT plasmid. (**b**) A western blot was used to analyze the changes in nuclear and cytoplasmic PPARα in the control and LRRK2-overexpressed HepG2 cells after 0 or 400 µM PA treatment for 2 h. Lamin A was a loading control for nuclear protein, and α-Tubulin was a loading control for cytoplasmic protein. Vector: vector control.

In order to investigate the mechanism of how LRRK2 regulates CPT1A in HepG2 cells, we analyzed upstream proteins from CPT1A by western blots. It has been shown that LRRK2 enhances the activation of AMPK to regulate autophagy [47]. Therefore, we examined whether LRRK2 also regulates the activity of AMPK in HepG2 cells. The results showed that the phosphorylated Thr172 of AMPK was increased in the LRRK2-overexpressed HepG2 cells (Figure 5a) The PPARα was up-regulated in LRRK2-overexpressed HepG2 cells (Figure 5a). The nuclear PPARα was increased after 400 μM PA treatment in LRRK2-overexpressed HepG2 cells compared to control cells (Figure 5b).

### 2.6. LRRK2 Suppressed the Levels of TNFα in HepG2 Cells after PA Treatment

**Figure 6:**
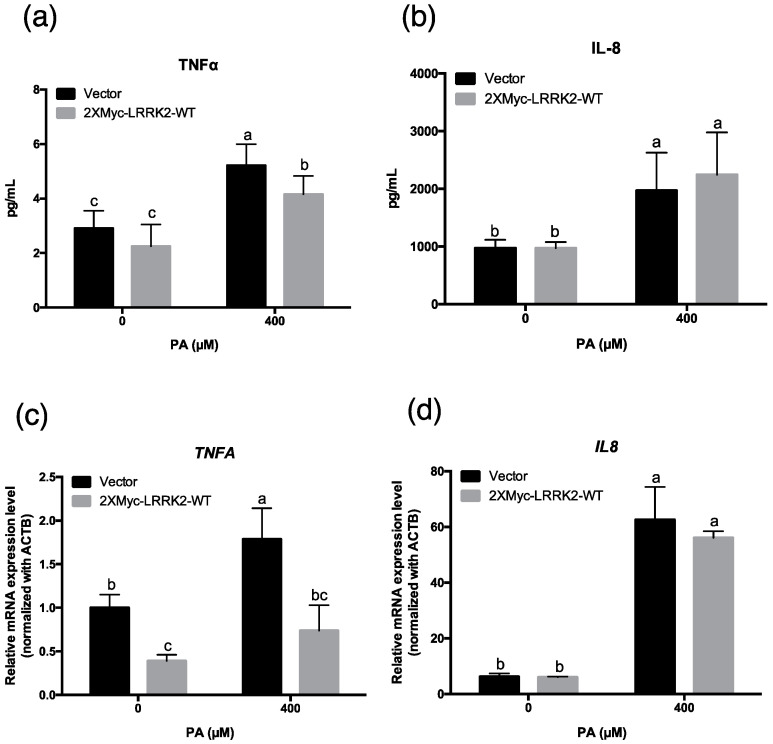
LRRK2 suppressed the levels of tumor necrosis factor α (TNFα) in HepG2 cells after PA treatment. (**a**) After vehicle or 400 μM PA treatment for 24 h, the secreted TNFα (*n* = 8 for each group) and (**b**) IL−8 from the vector control and LRRK2-overexpressed HepG2 cells were measured using an ELISA assay (*n* = 8 for each group). (**c**) Real-time PCR analysis of the mRNA levels of *TNFA* (*n* = 3 for each group) and (**d**) *IL8* (*n* = 3 for each group) in vector control and LRRK2-overexpressed HepG2 cells after treatment with vehicle or 400 μM PA for 24 h. Data were indicated as mean ± SD. Two-way ANOVA, *p* ≤ 0.05. Groups with no significant difference labeled with a common letter. Vector: vector control.

Previous studies indicate that the overexpression of CPT1A can suppress the released pro-inflammatory cytokines after treatment with free fatty acids [30,48]. In order to determine whether LRRK2 contributes to suppression of the pro-inflammatory cytokines released by HepG2 cells exposed to PA, we harvested the medium from the control and LRRK2-overexpressed HepG2 cells after treatment with PA for 24 h. The concentrations of extracellular TNFα of both vector control and LRRK2-overexpressed HepG2 cells were increased (*p* < 0.05) by 400 μM PA treatment (Figure 6a). However, compared to the control group, overexpression of LRRK2 decreased (*p* < 0.05) the concentration of extracellular TNFα, indicating that LRRK2 may suppress the TNFα induced by PA. In addition, our results indicated that overexpression of LRRK2 produced no changes in the concentration of IL-8 induced by PA (Figure 6b). We also analyzed the mRNA levels of *TNFA* and *IL8* in control and LRRK2-overexpressed HepG2 cells after treatment with PA. Overexpression of LRRK2 reduced (*p* < 0.05) the mRNA expression of *TNFA* compared to the vector control (Figure 6c). There was no change in the levels of *IL8* mRNA expression (Figure 6d). These data suggest that overexpression of LRRK2 suppressed the levels of TNFα induced by PA.

## 3. Discussion

Research on LRRK2 is mainly focused on the central nervous system and immune system, due to the association with Parkinson’s disease, inflammatory bowel disease, and leprosy [35,36,37]. However, the phenotype of LRRK2 knockout rodents indicates that fat is abnormally accumulated in the liver and kidney [38,39]. These studies imply that LRRK2 may be functional in the liver, particularly connected to lipid metabolism. According to previous studies, the expression of LRRK2 in liver is still controversial [49,50,51]. Our data showed that both the mRNA and protein of LRRK2 were detectable in mouse liver by real-time PCR and western blot analyses. In addition, LRRK2 is also expressed in hepatic cell lines. To elucidate the effects between LRRK2 and lipid metabolism in the liver, we measured the levels of LRRK2 in the livers of NAFLD mice after feeding a high-fat diet. Consistent with previous studies [42,43], the results showed the histological appearances of steatosis and steatohepatitis in the livers by high-fat diet feeding for 16 weeks. Increased mRNA levels of the pro-inflammatory cytokines, *Tnfa* and *Il1b*, confirmed that inflammation occurred in the livers of NAFLD mice. We also found that LRRK2 was down-regulated in the livers of NAFLD mice. However, mice fed with a high-fat diet combined with the consumption of high fructose/glucose for 8 weeks have an activated LRRK2-related pathway in livers, as analyzed by gene set enrichment [52]. The different NAFLD models or various time points during the development of NAFLD may result in the different pathophysiological status. Monitoring the change of LRRK2 level during the development of NAFLD in various models will be required to solve the controversial observations.

Current data showed that PA treatment led to a decrease of LRRK2 in HepG2 cells; however, OA had no effect on the expression of LRRK2, suggesting that the down-regulation of LRRK2 only occurs after treatment with a particular fatty acid. The effects on lipid metabolism caused by PA are distinct from OA [17,53]. Compared to PA, OA treatment increases intracellular TG levels [53,54]. The intracellular free fatty acids can be converted to TG and form lipid droplets [20]. Treatment with OA leads to the formation of TG-enriched lipid droplets and PA treatment yields less TG-enriched lipid droplets in cells [55,56]. OA increases the rate of fatty acid oxidation through sirtuin 1 and peroxisome proliferator-activated receptor gamma co-activator 1 α [57]. OA has little effect on apoptosis; however, PA leads to apoptosis and mitochondrial dysfunction [17,53,54,58]. Unlike OA, PA activates NF-κB signaling and induces the secretion of pro-inflammatory cytokines, such as TNFα and IL−8 [14]. In addition, exposure to PA leads to the impairment of fatty acid oxidation [16,17]. Therefore, the decrease in LRRK2 may be correlated with the different responses in the cells exposed to different fatty acids. 

Current data showed that LRRK2 decreased the levels of NEFA induced by PA, suggesting that LRRK2 may function to regulate β-oxidation and thus promote catabolism of NEFA. The LRRK2 positively regulated β-oxidation-related proteins, such as CPT1A, PPARα, and AMPK [23]. Previous studies show that knockout of LRRK2 results in the accumulation of fat in the livers and kidneys of rodents [38,39]. Our data indicated that LRRK2 was involved in the increase of CPT1A to promote β-oxidation. Therefore, the absence or extensive reduction in LRRK2 would reduce lipid catabolism. The steatosis in the livers and kidneys in LRRK2 knockout mice may be as a result of the diminished β-oxidation regulated by LRRK2.

The intracellular NEFA can be esterified and converted to TG stored in lipid droplets; this has been considered an adaptive and protective response to prevent lipotoxicity [20,55]. The free fatty acids accumulated within cells can be converted to ceramide and diacylglycerol [20]. The ceramide and diacylglycerol are lipotoxic lipids that can cause oxidative stress, inflammation, and liver damage associated with the progression of NAFLD [20,59]. The intracellular free fatty acids can also be transported into mitochondria and generate energy through β-oxidation [23]. Previous studies indicate that the promotion of β-oxidation via overexpression of CPT1A suppresses the concentration of pro-inflammatory cytokines released by cells after treatment with free fatty acids [30,31]. Our data showed that the over expression of LRRK2 suppressed TNFα induced by PA. Previous studies indicate that PA leads to increased ceramide in cells and livers [60,61,62,63]. Ceramide is a sphingolipid associated with oxidative stress and inflammation that is increased in the livers of NASH patients [64]. Inhibition of ceramide synthesis improves the steatosis and fibrosis in the livers of NAFLD rats [65]. The ceramide level is elevated in the brains from the LRRK2 knockout compared to wild-type mice [40]. Previous studies indicate that LRRK2 is involved in pro-inflammation in vivo and in vitro [35,66,67]. Thus, the anti-inflammatory effect of LRRK2 may not be a direct effect. We hypothesized that the anti-inflammatory effect of LRRK2 may be through the promotion of β-oxidation and decreased lipotoxic lipids.

In conclusion, we observed that LRRK2 was down-regulated in the livers from NAFLD mice and a PA-treated human hepatic cell line. The LRRK2 was involved in the regulation CPT1A to enhance β-oxidation, and may contribute to the suppression of inflammation induced by PA. The increase of CPT1A by LRRK2 was led by the activation of AMPK and PPARα. We conclude that the down-regulation of LRRK2 in the livers of NAFLD mice may result from the decrease of the catabolism of NEFA. Therefore, the induction of LRRK2 or increase in the activity of LRRK2 may be a strategy to improve NAFLD.

## 4. Materials and Methods

### 4.1. Animals

Male C57BL/6JNarl mice were provided by the National Laboratory Animal Center, National Applied Research Laboratories, Taipei, Taiwan. The mice were kept at 20−22 °C under a 12 h light/dark cycle. Food and water were given ad libitum throughout the experiments. The 8-week-old mice were randomly divided into two groups; one was fed a chow diet and one was fed a high-fat diet with 60% kcal as fat (D12492, Research Diets, Inc., New Brunswick, NJ, USA). After feeding the diets for 16 weeks, mice were sacrificed using CO_2_. Mouse livers were obtained immediately after sacrificing and divided into several portions for further analyses. Samples for protein and RNA analyses were snap-frozen in liquid nitrogen and then stored at −80 °C. Specimens for histological analyses were fixed in 10% formaldehyde and then embedded in paraffin. The animal experiments were approved by the Institutional Animal Care and Use Committee of National Taiwan University (NTU105-EL-00073).

### 4.2. Histological Analyses

The paraffin-embedded specimens were sliced at 4 μm using a microtome. The sections were stained with H&E to observe the pathological morphology of mouse livers. For immunohistochemistry (IHC) analysis, the procedure was described previously [68]. Briefly, the sliced sections reacted with anti-LRRK2 antibody (1/200, ab133474, Abcam, Cambridge, UK) as primary antibody. The detection of the signal was conducted using a horseradish peroxidase diaminobenzidine system (Agilent Technologies, Inc., Santa Clara, CA, USA).

### 4.3. RNA Extraction and Real-Time PCR Analyses

Total RNA of mouse liver and HepG2 cells was extracted using GENEzol^TM^ Reagent (Geneaid Biotech, Ltd., New Taipei City, Taiwan). The RNA concentration was measured at 260/280 nm using a NanoDrop^TM^ One (Thermo Fisher Scientific, Waltham, MA, USA). For each sample, 4 μg of RNA was treated with TURBO^TM^ DNase (Thermo Fisher Scientific, Waltham, MA, USA). The DNase-treated total RNA was used to generate cDNA using a High-Capacity cDNA Reverse Transcription Kit (Thermo Fisher Scientific, Waltham, MA, USA). The cDNA was utilized to conduct real-time PCR using SensiFast^TM^ SYBR No-ROX Kit (Bioline, London, UK) via CFX96^TM^ Real-Time System (Bio-Rad Laboratories, Inc., Berkeley, CA, USA). Real-time PCR was performed using the following program: two minutes at 95 °C for polymerase activation, 40 cycles at 95 °C for five seconds for denaturation and 60 °C for 30 s for annealing/ extension. The primer sets are listed in Table 1.

### 4.4. Protein Extraction and Western Blot

Liver tissue was ground on ice using a Teflon pestle in an eppendorf with RIPA lysis buffer (EMD Millipore, Waltham, MA, USA), supplemented with protease and phosphatase inhibitors (Thermo Fisher Scientific, Waltham, MA, USA). The lysed, homogenized liver samples were centrifuged after 30 min (10,000× *g* was for 30 min at 4 °C). The supernatant fraction was kept as the liver protein sample. The concentration of the protein was determined using the BCA protein assay (Thermo Fisher Scientific, Waltham, MA, USA). The cell lines were lysed and centrifugated as indicated for mouse livers. The concentration of cell protein was determined using the BCA assay as well. For Western blots, equal amounts of sample proteins were diluted with 40 mM Tris (pH 6.8), 1% dodecyl sodium sulfate, 5% glycerol, 0.0003% bromophenol blue, and 0.05M DTT. Electrophoresis was performed using 6% to 10% SDS-PAGE with a running buffer composed of 25 mM Tris, 190 mM Glycine, and 0.1% SDS at 80 V, until the dye of the sample buffer reached the bottom of the gel. Before transfer, gels were soaked in transfer buffer composed of 25 mM Tris, 190 mM Glycine, and 20% methanol for 20 min. Proteins were transferred from gels to methanol-activated PVDF membranes (PerkinElmer, Inc., Waltham, MA, USA) in transfer buffer at 200 mA for 2 h. After transfer, PVDF membranes were soaked in a blocking buffer composed of 25 mM Tris (pH7.4), 150 mM NaCl, 0.1% Tween 20, and 5% skim milk with gentle shaking for 1 h. Membranes were incubated with primary antibodies diluted in TBST buffer, composed of 25 mM Tris (pH 7.4), 150 mM NaCl, and 0.1% Tween 20 overnight at 4 °C. After the incubation with primary antibodies, PVDF membranes were washed using TBST buffer three times for 10 min each. Then, the membranes were incubated with secondary antibodies diluted in TBST buffer at room temperature for 1 h. After washing in TBST buffer, PVDF membranes were incubated with Clarity^TM^ Western ECL Substrate (Bio-Rad Laboratories, Inc., Santa Clara, CA, USA) and detected using the ChemiDoc Touch Imaging System (Bio-Rad Laboratories, Inc., Santa Clara, CA, USA). The quantitation of bands was conducted using Image Lab software (Bio-Rad Laboratories, Inc., Santa Clara, CA, USA), and the relative levels of particular proteins in samples were normalized with α-tubulin. The antibodies used for Western blot were anti-LRRK2 (1/1000, ab133474, Abcam, Cambridge, UK); anti-CPT1A (1/1000, ab128568, Abcam, Cambridge, UK); anti-PPARα (1/1000, sc-9000, Santa Cruz Biotechnology Inc., Dallas, TX, USA); anti-phospho-AMPK (Thr 172) (1/1000, #2535, anti-AMPK (1/1000, #2532,) and; anti-Lamin A/C (1/1000, #2032, Cell Signaling Technology, Inc., Danvers, MA, USA); anti-α-Tubulin (1/1000, ab52866, Abcam, Cambridge, UK); anti-β-Actin (1/1000, sc-47778, Santa Cruz Biotechnology Inc., Dallas, TX, USA); anti-rabbit IgG (1/5000, #7074); and anti-mouse IgG (1/5000, #7076, Cell Signaling Technology, Inc., Danvers, MA, USA).

### 4.5. Cell Culture and Fatty Acid Treatment

Hep3B and PLC5 cells were gifts from Professor Shiou-Hwei Yet in Graduate Institute of Microbiology, College of Medicine, National Taiwan University. HepG2, Hep3B, and PLC5 cells were grown in Dulbecco’s modified Eagle medium (DMEM) supplemented with 10% fetal bovine serum (Thermo Fisher Scientific, Waltham, MA, USA) and 1% penicillin- streptomycin- amphotericin B Solution (Biological Industries, Kibbutz Beit Haemek, Israel). Prior to the treatment with fatty acids, 6 × 10^5^ cells were seeded on 6-well plates and incubated overnight in a cell incubator with 5% CO_2_ in air at 37 °C. Palmitic acid (PA) (Cayman Chemical Company, Ann Arbor, MI, USA) and oleic acid (OA) (Cayman Chemical Company, Ann Arbor, MI, USA) were both dissolved in ethanol (Sigma-Aldrich Corporation, St. Louis, MO, USA), and the dissolved PA or OA was aliquoted and stored at −20 °C. The PA or OA was conjugated with 1% BSA with low fatty acid, low endotoxin, and low IgG (US Biological Life Sciences, Swampscott, MA, USA), in complete DMEM medium prior to treating cells. The BSA-conjugated fatty acids were added to the wells of 6-well plates for the indicated time.

### 4.6. Knockdown of LRRK2 in HepG2 Cells

The lentivirus harboring the shRNA of scramble control or LRRK2 were purchased from RNA Technology Platform and Gene Manipulation Core, Academia Sinica, Taiwan. The target sequence of scramble and LRRK2 are as follows: scramble = CCTAAGGTTAAGTCGCCCTCG; LRRK2 = CCCAGGATGTTGGAAATGATT. The HepG2 cells were seeded at 2 × 10^5^ cells on 6-well plates and were incubated overnight. Before lentiviral infections, medium was replaced by growth media (complete DMEM medium with 8 μg / mL polybrene (Sigma-Aldrich Corporation, St. Louis, MO, USA), and finally the viruses were added. Media were replaced with fresh growth medium after viral incubation for 24 h. The analyses of western blots, triglyceride (TG), and NEFA were performed within 48 h after lentiviral infections.

### 4.7. Overexpression of LRRK2

The 2XMyc-LRRK2-WT plasmid harboring a human LRRK2 gene was a gift from Mark Cookson (Addgene plasmid #25361; http://n2t.net/addgene:25361; RRID: Addgene_25361) [44]. The sequence of the LRRK2 coding region was confirmed by DNA sequencing. To generate a control plasmid, 2XMyc-LRRK2-WT plasmid was digested with NotI-HF and KpnI-HF (New England Biolabs, Rowley, MA, USA) to separate the coding region of LRRK2 from the vector backbone. The fragment of 4187 bp was isolated via DNA electrophoresis on 0.8% agarose gel (Vivantis Technologies, Selangor, Malaysia), and purified using a QIAEX II Gel Extraction Kit (Qiagen, Hilden, Germany). The isolated fragment was blunted by T4 DNA polymerase (New England Biolabs, Rowley, MA, USA) and ligated using T4 DNA ligase (New England Biolabs, Rowley, MA, USA); the end product was used as the control plasmid for the 2XMyc-LRRK2-WT plasmid. Prior to DNA transfection, 4 × 10^5^ HepG2 cells were seeded on a 6-well plate and incubated overnight. The DNA transfection was performed using PolyJet^TM^ (SignaGen Laboratories, Montgomery, MD, USA), and its manufacturer’s guidelines were followed during the process of transfection.

### 4.8. Measurement of TG and NEFA

After the treatment with PA or OA for 24 h, cells were washed twice with PBS. The cells were scraped with PBS using cell scraper and harvested into glass tubes. One-tenth of the volume of cell lysate was obtained to extract protein for protein quantification. To extract intracellular lipids, one part of cell lysate was added to four parts of chloroform/methanol (2/1, *v/v*) [69]. Then, the cell lysates were vortexed vigorously for one minute. After incubating on ice for two hours, cell lysates were centrifuged at 1650× *g* at 4 °C for 10 min. After centrifugation, the bottom phase was transferred into a new glass tube using a glass Pasteur pipet. The fractions of bottom phase were dried using nitrogen gas at 37 °C. The lipid samples were dissolved using isopropanol/Nonidet P-40 (9:1, *v/v*) solution [70]. The measurement of TG and NEFA was performed using TRIGS and NEFA detection kits (Randox Laboratories, County Antrim, UK), respectively; the signals for TG and NEFA were detected using an ELISA reader at 500 nm and 550 nm, respectively. Protein concentrations of the samples were measured using the BCA assay. To compare the relative levels of various samples, the concentration of TG or NEFA of each sample was normalized with its protein concentration. 

### 4.9. Measurement the Activity of Fatty Acid Oxidation

First, 1 × 10^6^ cells were washed with PBS before harvesting cell lysates. The extraction of cell lysates was conducted using the cell lysis solution provided in the fatty acid oxidation (FAO) assay kit (Biomedical Research Service Center, New York, NY, USA). After obtaining the cell lysates, the protein concentration of samples was determined using the BCA assay. The samples were diluted with the cell lysis solution to 2 mg/mL to measure the activity of fatty acid oxidation. The procedures followed the manufacturer’s instructions, and the signals were detected using an ELISA reader at 492 nm. To compare the relative levels of various samples, the activity of fatty acid oxidation of each sample was normalized with values from control groups.

### 4.10. Extraction of Cytoplasmic and Nuclear Proteins

Next, 1 × 10^6^ cells were washed with PBS before extracting the cytoplasmic and nuclear proteins. The isolation of cytoplasmic and nuclear proteins was performed using EpiXtract^TM^ Nuclear Protein Isolation Kit II (nucleic acid-free) (Enzo Life Sciences Inc., New York, NY, USA). After extracting cytoplasmic and nuclear proteins, the BCA assay was utilized to determine the protein concentrations. Equal amounts of proteins were used for western blot analysis.

### 4.11. ELISA Analysis of TNFα and Interleukin 8 Concentrations

The medium for ELISA measurements was obtained after PA or vehicle treatment. To remove the debris in the medium, it was centrifugated at 150× *g* for 5 min and the supernatant fraction was retained. Human TNF alpha ELISA Ready-Set-Go^TM^ and IL-8 ELISA Ready-Set-Go^TM^ kits (Thermo Fisher Scientific, Waltham, MA, USA) were utilized to measure the concentration of extracellular TNFα and interleukin 8 (IL-8) in the medium, respectively. The signals were detected using an ELISA reader at 570 nm.

### 4.12. Statistical Analysis

Data were analyzed using GraphPad Prism (GraphPad Software, Inc., CA, USA) and represented as mean ± SEM or mean ± SD as indicated. A paired *t* test, one-way ANOVA, and two-way ANOVA followed by Tukey’s multiple comparison test were used for group comparisons. A *p* value ≤ 0.05 was considered statistically different.

## Figures and Tables

**Table 1:** Primer sets for real-time PCR.

Target Gene	Primer Sequence	Reference Sequence
Mouse *Fasn*	F: GGAGGTGGTGATAGCCGGTAT R: TGGGTAATCCATAGAGCCCAG	NM_007988.3
Mouse *Srebp1c*	F: GGAGCCATGGATTGCACATT R: GGCCCGGGAAGTCACTGT	NM_001358314.1
Mouse *Acc*	F: TAATGGGCTGCTTCTGTGACTC R: CTCAATATCGCCATCAGTCTTG	NM_133360.2
Mouse *Pparg*	F: TTGCTGTGGGGATGTCTCAC R: AACAGCTTCTCCTTCTCGGC	NM_001127330.2
Mouse *Tnfa*	F: CCACGTCGTAGCAAACCAC R: TTGTCCCTTGAAGAGAACCTG	NM_013693.3
Mouse *Il1b*	F: GCAGTGGTTCGAGGCCTAAT R: GCTGCTTCAGACACTTGCAC	NM_008361.4
Mouse *Lrrk2*	F: ATGGAGTTGGCCTCCAAAGG R: GATCCCGTAGTCCGCAATCT	NM_025730.3
Mouse *Ppia*	F: AGGATTCATGTGCCAGGGTG R: GATGCCAGGACCTGTATGCT	NM_008907.2
Human *TNFA*	F: AGCCTCTTCTCCTTCCTGAT R: AAGATGATCTGACTGCCTGG	NM_000594.4
Human *IL8*	F: CCAGGAAGAAACCACCGGA R: GAAATCAGGAAGGCTGCCAAG	NM_000584.4
Human *ACTB*	F: GAAGATCAAGATCATTGCTCCTC R: CTAAGTCATAGTCCGCCTAGAAG	NM_001101.5

## Abbreviations

1. AMPK	AMP-activated protein kinase
2. CPT1A	Carnitine palmitoyltransferase 1A
3. H&E	Hematoxylin and eosin
4. IHC	Immunohistochemistry
5. IL-8	Interleukin-8
6. LRRK2	Leucine-Rich Repeat Kinase 2
7. NAFLD	Nonalcoholic fatty liver disease
8. NASH	Nonalcoholic steatohepatitis
9. NEFA	Non-esterified fatty acid
10. NF-κB	Nuclear factor kappa B
11. OA	Oleic acid
12. PA	Palmitic acid
13. PPARα	Peroxisome proliferator-activated receptor α
14. shRNA	Short hairpin RNA
15. TG	Triglyceride
16. TNFα	Tumor necrosis factor α
